# Essential APSES Transcription Factors for Mycotoxin Synthesis, Fungal Development, and Pathogenicity in *Aspergillus flavus*

**DOI:** 10.3389/fmicb.2017.02277

**Published:** 2017-11-20

**Authors:** Guangshan Yao, Feng Zhang, Xinyi Nie, Xiuna Wang, Jun Yuan, Zhenhong Zhuang, Shihua Wang

**Affiliations:** Key Laboratory of Pathogenic Fungi and Mycotoxins of Fujian Province, Key Laboratory of Biopesticide and Chemical Biology of Education Ministry, School of Life Sciences, Fujian Agriculture and Forestry University, Fuzhou, China

**Keywords:** APSES transcription factor, aflatoxin, *Aspergillus flavus*, fungal development, cyclopiazonic acid

## Abstract

Aflatoxins are a potent carcinogenic mycotoxin and has become a research model of fungal secondary metabolism (SM). Via systematically investigating the APSES transcription factors (TFs), two APSES proteins were identified: AfRafA and AfStuA. These play central roles in the synthesis of mycotoxins including aflatoxin and cyclopiazonic acid, and fungal development and are consequently central to the pathogenicity of the aflatoxigenic *A. flavus*. Loss of AfRafA not only dramatically suppressed aflatoxin cluster expression, subsequently reducing toxin synthesis both *in vitro* and *in vivo*, but also impaired conidia and sclerotia development. More importantly, aflatoxin biosynthesis as well as conidia and sclerotia development were fully blocked in Δ*AfStuA*. In addition, our results supported that AfStuA regulated the aflatoxin synthesis in an AflR-dependent manner. Intriguingly, it was revealed that AfRafA and AfStuA exert an antagonistic role in the regulation of biosynthesis of cyclopiazonic acid. In summary, two global transcriptional regulators for fungal development, mycotoxin production, and seed pathogenicity of the *A. flavus* system have been established. The two novel regulators of mycotoxins are promising targets for future plant breeding and for the development of fungicides.

## Introduction

Genomic and metabolic investigations revealed that filamentous fungi host a large arsenal of secondary metabolites, many of which exhibited excellent bioactive activity and thus have been applied as therapeutic drugs (e.g., penicillium and lovastatin) in pharmaceutical development. However, a number of secondary metabolites (SM) called mycotoxins (including aflatoxin, fumonisins, ochratoxin, trichothecenes, and cyclopiazonic acid) that widely exist on crops and agricultural products are severely threatening both human and animal health. Among these SM, Aflatoxin (AF) (and particularly AFB1) was ranked as the most carcinogenic natural product that has been discovered (Squire, 1981; Lee and Adams, 1994). Ingestion of a high concentration of AFB1 causes acute aflatoxicosis, regularly killing humans in developing countries (Probst et al., 2007) and animals in the United States (Newman et al., 2007). However, long-term and low-dosage uptake of AFB1 by either humans or animals may induce hepatocellular carcinoma (HCC) through a conversion by cytochrome P450 into AFB1 adducts (Kew, 2013). Moreover, a systematic meta-analysis of more than four billion people worldwide has shown that aflatoxin interacts with the hepatitis B virus (HBV), thus synergistically inducing HCC development (Liu et al., 2012). To eliminate negative effect by aflatoxins on human and animals, long-term and efficacious control strategies for both its synthesis and the producing fungi are urgently required. To this end, it is significant to comprehensively and deeply understand the molecular mechanism for the regulation of aflatoxin production.

Aflatoxins are polyketide chemicals and their synthesis require a variety of enzymatic reactions that have been extensively studied over the past 20 years. The aflatoxin cluster contains about 30 genes and their specific roles in aflatoxin synthesis have almost been clarified; background information can be accessed in multiple reviews on the topic (Bhatnagar et al., 2006; Amaike and Keller, 2011; Roze et al., 2013). However, the regulatory mechanisms for aflatoxin synthesis still remain poorly understood, as only few aflatoxin regulators have yet been identified (such as the pathway-specific TFs AflR and AflS, and the global regulator LaeA and VeA). Despite previous reports of a close association of SM biosynthesis with fungal asexual (conidiation) as well as sexual development in filamentous fungi (Calvo et al., 2002; Bhatnagar et al., 2006; Amare and Keller, 2014), both the physiological significance and the molecular basis for this connection remain largely unknown. Conidia are a major way for dispersion and propagation of *A. flavus* as well as for other filamentous fungi and a previous report showed that aflatoxin was significantly concentrated in the conidia (Wicklow and Shotwell, 1983). Furthermore, mutants with abolished or partly defective conidiation constantly produced no or lower levels of aflatoxin or other mycotoxins (Calvo et al., 2002; Amare and Keller, 2014). Among those, the most representative were the SM global regulators LaeA and VeA. Loss of LaeA or VeA resulted in defects in both conidial development and aflatoxin synthesis in *A. flavus* (Kale et al., 2008; Amaike and Keller, 2009; Chang et al., 2012a), as well as deoxynivalenol (DON) biosynthesis in *Fusarium graminearum* (Jiang et al., 2011). The master transcription factor MtfA was reported to not only strongly affect fungal development, but also to regulate the biosynthesis of aflatoxin in *A. flavus* (Zhuang et al., 2016), sterigmatocystin (the penultimate precursor to aflatoxin) in *A. nidulans* (Ramamoorthy et al., 2013), and gliotoxin in *A. fumigatus* (Smith and Calvo, 2014). Therefore, we propose that the regulators, with central roles in fungal development, are potentially involved in controlling the biosynthesis of aflatoxin and other SMs.

The APSES (Asm1p, Phd1p, Sok2p, Efg1p, and StuAp) TFs had been demonstrated to be key regulators of fungal development and have been conserved from yeast to plant or human pathogens, as previously reviewed (Zhao et al., 2015). In *Saccharomyces cerevisiae*, the five APSES proteins Phd1, Sok2, Xbp1, Swi4, and Swi6, have previously been demonstrated to control fundamental biological processes, including pseudohyphal growth, cell growth, cell division, cell metabolism, and cell cycle control (Gimeno and Fink, 1994; Ward et al., 1995; Koch et al., 1996; Miles et al., 2013). The two APSES TFs Efg1 and Efh1 from the human pathogens *Candida albicans* and *C. parapsilosis*, respectively, play pivotal roles for fungal morphogenesis and metabolism, concentric-smooth phenotype switching, biofilm formation, and virulence (Stoldt et al., 1997; Doedt et al., 2004; Noffz et al., 2008; Connolly et al., 2013). Both yeast Phd1 and Sok2, *Candida* Efg1 and Efh1 are orthologs of StuA that originated from filamentous fungi and all of these contain a conserved KilA-N domain. Increasing evidence demonstrates that (as a canonical APSES TF) StuA plays both conserved and central roles in the asexual development of fungi (conidiation or sporulation) as well as in the morphogenesis of diverse filamentous fungi (Zhao et al., 2015). In *A. nidulans*, StuA controls the expression of both critical developmental regulatory genes *brlA* and *abaA* and cell cycle genes by directly binds the conserved motif (A/TCGCGT/ANA/C), to direct fungal developmental program (Dutton et al., 1997). In addition, StuA also regulates the cell wall biogenesis by repressing *fksA* (encoding β-1,3-glucan synthase) expression during asexual development (Park et al., 2014). Recently, a putative APSES transcription factor has also been demonstrated to be required for growth and development in *A. nidulans* (Lee et al., 2013). However, in addition to StuA, the regulatory roles of APSES TFs remain poorly understood in the biosynthesis of the secondary metabolism. Especially, none of the APSES TFs have so far been identified to play a role in fungal development and aflatoxin synthesis in the aflatoxin-producing fungus *A. flavus*.

All *Aspergillus* produce conidia as a major way of dispersion and propagation, and thus provide a well-established model to study spatial and temporal regulation of conidiation (Ni et al., 2010). The C_2_H_2_ zinc finger transcription factor BrlA functions as an essential activator for conidiation in *Aspergillus*, its loss of function mutant lead to a block of asexual development (Adams et al., 1988; Ni et al., 2010). AbaA, as a switch, controls the conidiation (Andrianopoulos and Timberlake, 1994). WetA, seems to function upstream of both BrlA and StuA, regulates conidial cell wall maturation (Marshall and Timberlake, 1991). Further, it has been proposed that BrlA→AbaA→WetA defines a central regulatory cascade, cooperatively activate genes responsible for conidiation in *Aspergillus* (Ni et al., 2010). *A. flavus* also produce another reproductive and resistant structure sclerotia. And, it was suggested an association between fungal secondary metabolism and sclerotial development (Calvo and Cary, 2015). So far, three genes have been demonstrated to regulate sclerotial development including *sclR, nsdC*, and *nsdD* (Jin et al., 2011; Cary et al., 2012).

In the present study, we systematically investigated all APSES TFs in the economically important pathogen *A. flavus* and our results showed that both AfRafA and AfStuA are essential for activating aflatoxin gene cluster expression and subsequent toxin synthesis, the development of conidia and sclerotia, as well as the colonization of plant seeds. Our evidence demonstrated that AfStuA partially controls aflatoxin synthesis via the pathway-specific transcription factor AflR. In addition, our results implied that AfStuA might indirectly regulate the aflatoxin synthesis by modulating glutamine metabolism. More interestingly, our data demonstrated that AfStuA and AfRafA play contrasting roles in the biosynthesis of cyclopiazonic acid.

## Materials and methods

### Strains and culture conditions

The uracil auxotrophic strain *A. flavus* PTSΔ*ku70*Δ*pyrG* (Chang et al., 2010) was used as recipient strain for gene deletion, and PTSΔ*ku70*Δ*pyrG:*: *AfpyrG* was used as wild type strain (WT). For the phenotype assay, all utilized *A. flavus* strains were point-inoculated onto Potato Dextrose Agar (PDA), YES (2% yeast extract, 6% sucrose, and 0.1% MgSO_4_), and Czapek–Dox agar (CA) and were cultured under either dark or light conditions at 29°C for 6 days. The colony diameters were measured from day 2 to day 6 and the growth rate was subsequently calculated. Three plugs for one plate were collected and 1 ml ddH_2_O was added. After vortexing, conidia were counted, using a hemocytometer. Sclerotia formation and cyclopiazonic acid (CPA) production were induced on Wickerham medium (WKM) following previously described methods (Chang et al., 2012b), and sclerotial plates were washed several times with 70% ethanol before visualization and counting.

### Determination of aflatoxin and CPA production via TLC and HPLC

The HPLC-grade AFB1 and CPA standards were purchased from Sigma (Sigma, Germany). The utilized *A. flavus* strains were pre-grown in YGT (0.5% yeast extract, 2% glucose, and 0.1% trace element solution) liquid and 0.25 g mycelia of all strains were transferred into YES or Glucose minimum media (GMM) +glutamine (5 mM) (to induce aflatoxin) and WKM (to induce CPA) liquid media, and stationary cultured at 29°C under dark conditions for 7 days. The cultures were collected via filtering and aflatoxin was extracted via chloroform. After drying overnight, 20 μl chloroform or 1 ml methanol were added, to re-dissolve these toxins for TLC or HPLC analysis, respectively. TLC analysis of aflatoxin was performed with the acetone: chloroform (1:9, v/v) solvent system, and results were displayed under ultraviolet activation at 365 nm. CPA was separated on the ethyl acetate: methanol: ammonium hydroxide (85:15:10) solvent system and displayed via adding Ehrlich's reagent as previously described (Chang et al., 2017). The HPLC analysis was conducted using Waters HPLC (Waters, USA) equipped with a MYCOTOX™ reversed-phase C18 column (4.6 × 250 mm) and a fluorescent detector. The column was equilibrated in the mobile phase (56:22:22, water: methanol: acetonitrile) at 42°C for 1 h, and the aflatoxin extracts dissolved in methanol were filtered and separated in a 100% mobile phase at a flow rate of 1.0 ml/min (15 min per sample). The AFB1 concentration of individual strains was counted by using calibration curves of AFB1.

### Gene deletion and complementation

Double-joint PCR was used to construct a gene deletion cassette, using the *pyrG* gene amplified from *A. fumigatus* as the selectable marker. All primers that were used to amplify the 5′- and 3′-flanks were listed in Table S1 with the *A. flavus* gDNA as template. The entire gene deletion cassette was amplified with specific primers, using the 5′- and 3′-flanks for gene *AFLA_008860, 0AFLA_020130, AFLA_046990, AFLA_076560, AFLA_132630*, and *pyrG* mix as template. The PCR products were transformed into the protoplasts of PTSΔ*ku70*Δ*pyrG*. Protoplast preparation and PEG-mediated fungal transformation were performed following previously described methods (Chang, 2008). For gene complementation, PCR products of native promoter and open reading frame for AfRafA and AfStuA, combined with plasmid pPTR1 containing the marker gene *ptrA*, were re-introduced into the protoplasts of their gene deletion strain. PCR was used to preliminary identify the positive transformants and results were then confirmed via Southern blot and RT-PCR. The probes for Southern blot analysis were amplified from WT genome via gene-specific primers, and probe labeling, hybridization and detection were performed with the DIG High Prime labeling kit (Roche). RT-PCR was performed with primers for AfRafA or AfStuA-specific to further verify PCR- and Southern blot-positive transformants. All primers used are listed in Table S1.

### RNA extraction, reverse transcription, and quantitative real-time PCR

All utilized *A. flavus* strains were inoculated into YES liquid media and pre-cultured for 24 h, and then transferred into fresh YES media for stationary culture at 29°C for 48 h. The mycelia were collected via filtering, and conidia and sclerotia collected from PDA and WKM media, respectively, were frozen in liquid nitrogen, and stored under −80°C conditions. RNA extraction and cDNA synthesis were performed using a RNA reagent and Kit following the protocols of the manufacture (TRIzol reagent, Biomarker Technologies, Beijing, China). The quality and integrity of RNA samples was determined using Nanodrop and Agilent 2100 bioanalyzer (Agilent Technologies, Palo Alto, CA), respectively, while the quantity was determined with a Qubit RNA assay kit (Life Invitrogen, USA). Real–time quantitative PCR (qPCR) was performed on a PIKO REAL Real-Time PCR System (Thermo Scientific, Inc.) as previously described (Wang et al., 2017). All utilized primers were listed in Table S1. The relative expression level of each assayed gene was calculated using the 2^−ΔΔCt^ method and the expression of actin was used as internal control.

### Peanut and maize seed infection

Peanut (*Arachis hypogaea*) and maize seeds (*Zea mays L*.) with high activity were used to measure the pathogenicity of all *A. flavus* strains following previously described methods, but with minor modification (Tsitsigiannis and Keller, 2006). Briefly, both peanut and maize seeds were prepared via testa removal. Cotyledons without the embryo were sterilized via 75% ethanol and 30 peanut or maize cotyledons were inoculated with 10^6^ conidia for all *A. flavus*, with the exception of Δ*AfStuA* for which hyphae were used for infection. The process of infection was performed under 29°C for 7 days. The *A. flavus*-infected seeds were collected into a tube and 2 ml ddH_2_O were added to wash conidia. The conidia were counted as described above and the weight of peanut or maize was normalized. Furthermore, aflatoxin was extracted as described above, and dissolved into methanol for HPLC analysis. Both the conidia and aflatoxin concentration were normalized according to the weight of peanut and maize seeds. The control was performed via addition of sterile ddH_2_O to replace the *A. flavus*.

### Statistical analysis

The significance of the data was tested using the Student's *t*-test. A *P*-value < 0.05 was considered as significant difference.

## Results

### APSES TFs in aflatoxigenic *A. flavus*

**Figure 1:**
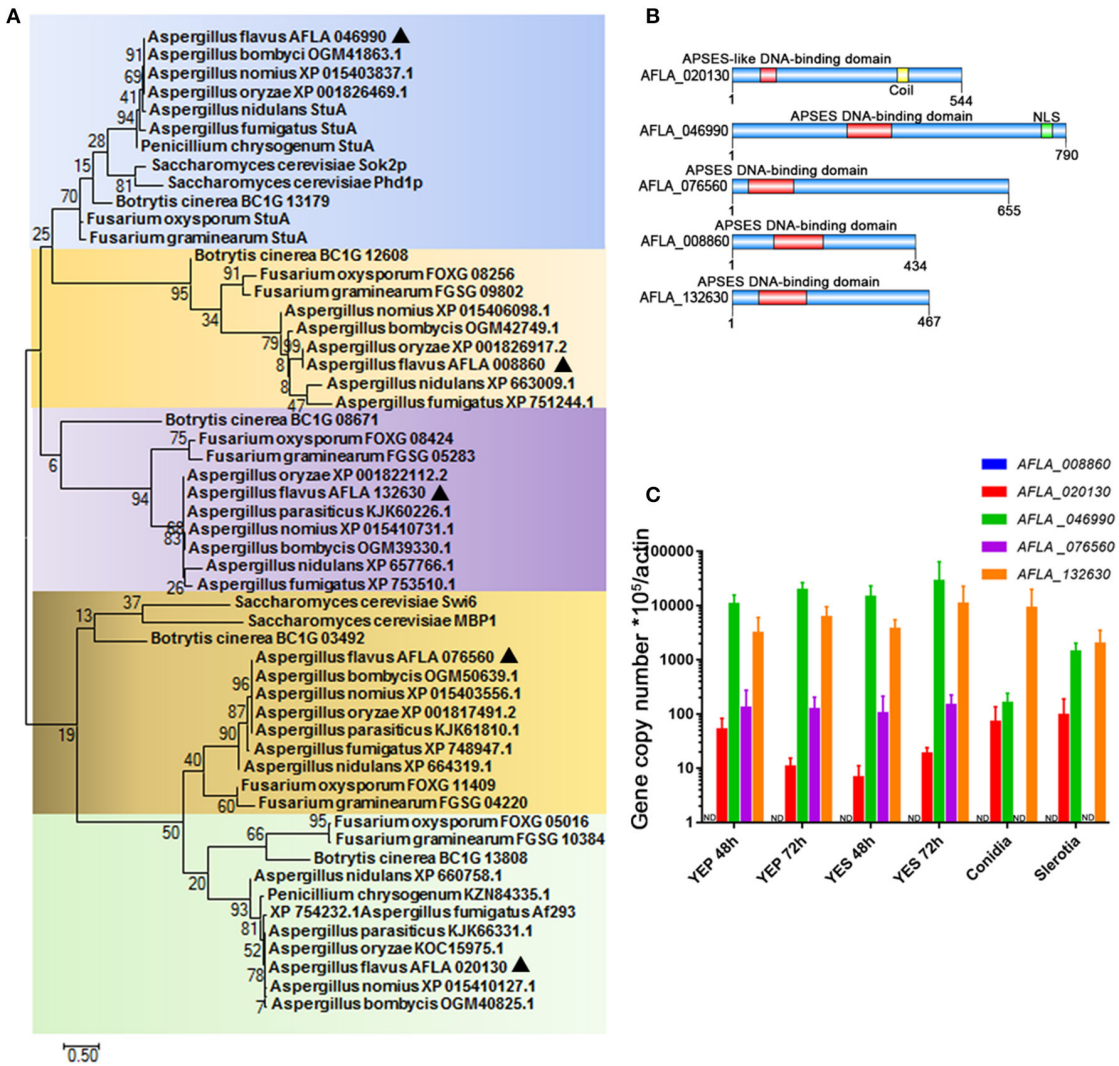
APSES transcription factor in *A. flavus*. **(A)** Phylogenetic analysis of the APSES protein orthologs AFLA_008860, AFLA_020130, AFLA_046990, AFLA_076560, and AFLA_132630. Both *A. flavus* APSES proteins and their orthologs were downloaded from the NCBI database, and sequence alignment and Maximum-likelihood tree were constructed using the software MEGA 7.0.21 (Kumar et al., 2016). **(B)** Schematic depiction of the APSES transcription factors (from top to bottom: AFLA_020130, AFLA_046990, AFLA_076560, AFLA_008860, and AFLA_132630) from *A. flavus* **(C)** Expression profile of APSES TFs encoding genes from *A. flavus* under various conditions (YEP, YES, conidia, and sclerotia).

The conserved regulators that play roles in fungal development program, are commonly involved in the control of SM synthesis in filamentous fungi. We therefore propose that those transcription factors that contain an APSES DNA-binding domain (which have previously been demonstrated to regulate the fungal development process in *S. cerevisiae* and fungal pathogens) may assume central roles in the synthesis of aflatoxin. While screening the genome of the major aflatoxin-producer *A. flavus* (http://fungi.ensembl.org/Aspergillus_flavus/Info/Index), we identified five proteins with a highly conserved APSES DNA binding domain in the N-terminal, and thus mark them as APSES transcription factors. These were AFLA_020130, AFLA_046990, AFLA_076560, AFLA_008860, and AFLA_132630. Phylogenetic analysis revealed that APSES transcription factor orthologs of *Aspergillus* were grouped and their phylogenetic relationship correlated well with species evolution (Figure 1A). Interestingly, AFLA_046990, AFLA_020130, and AFLA_076560 orthologs from *Aspergillus* species, which harbor the aflatoxin cluster include *A. flavus, A. oryzae, A. nomius*, and *A. bombycis*, were cluster into a branch, separate from their close relatives *A. nidulans* and *A. fumigatus*. So far, only the orthologs of AFLA_046990 Sok2p and Phd1p in yeast and StuA in several filamentous fungi have been identified. Via the *A. flavus* amino acid sequences of AFLA_046990 as query, we searched against the NCBI database using protein-protein BLAST and found that AFLA_046990 shared high similarity with *S. cerevisiae* Sok1, *A. nidulans* and *A. fumigatus* StuA, and *N. crassa* ASM1. In summary, this suggests AFLA_046990 as the homolog of StuA. Other APSES TFs and their orthologs have not been characterized, and even the orthologs of AFLA_008860 and AFLA_132630 were absent in *S. cerevisiae* and some filamentous fungi, suggesting that they might have originated via gene duplication in the later evolution events. The lengths of five APSES proteins from *A. flavus* ranged from 434 aa for AFLA_008860 to 790 aa for AFLA_046990 (Figure 1B). In contrast to AFLA_020130, four TFs shared a typical APSES domain (IPR003163) in their N-terminals, which is likely responsible for binding with the DNA motif. AFLA_020130 contains the APSES-like domain (IPR020683), which has been associated with an APSES domain in *N. crassa* (Aramayo et al., 1996). Prior to gene functional characterization, the expression profiles for their encoding genes were tested. A comprehensive analysis of the expression of genes *AFLA_008860, AFLA_020130, AFLA_046990, AFLA_008860*, and *AFLA_132630* was performed under various conditions. Expression levels of them in aflatoxin inducing media (YES), YES-like aflatoxin non-inducing media (YEP), as well as in conidia and sclerotia were determined by real-time quantitative PCR (qPCR) (Figure 1C). A clear result showed that *AFLA_046990* displayed the highest mRNA level in all assayed conditions and was slightly induced under YES compared to YEP, implying its important roles in fungal development and aflatoxin synthesis. Furthermore, both *AFLA_046990* and *AFLA_076560* showed low expression in both conidia and sclerotia in comparison to vegetative mycelia. In contrast, other APSES genes such as *AFLA_020130* and *AFLA_132630* showed constitutive expression under a variety of conditions. However, expression of *AFLA_008860* could not be detected under any assayed condition.

### AfStuA is essential and AfRafA is required for aflatoxin synthesis

**Figure 2:**
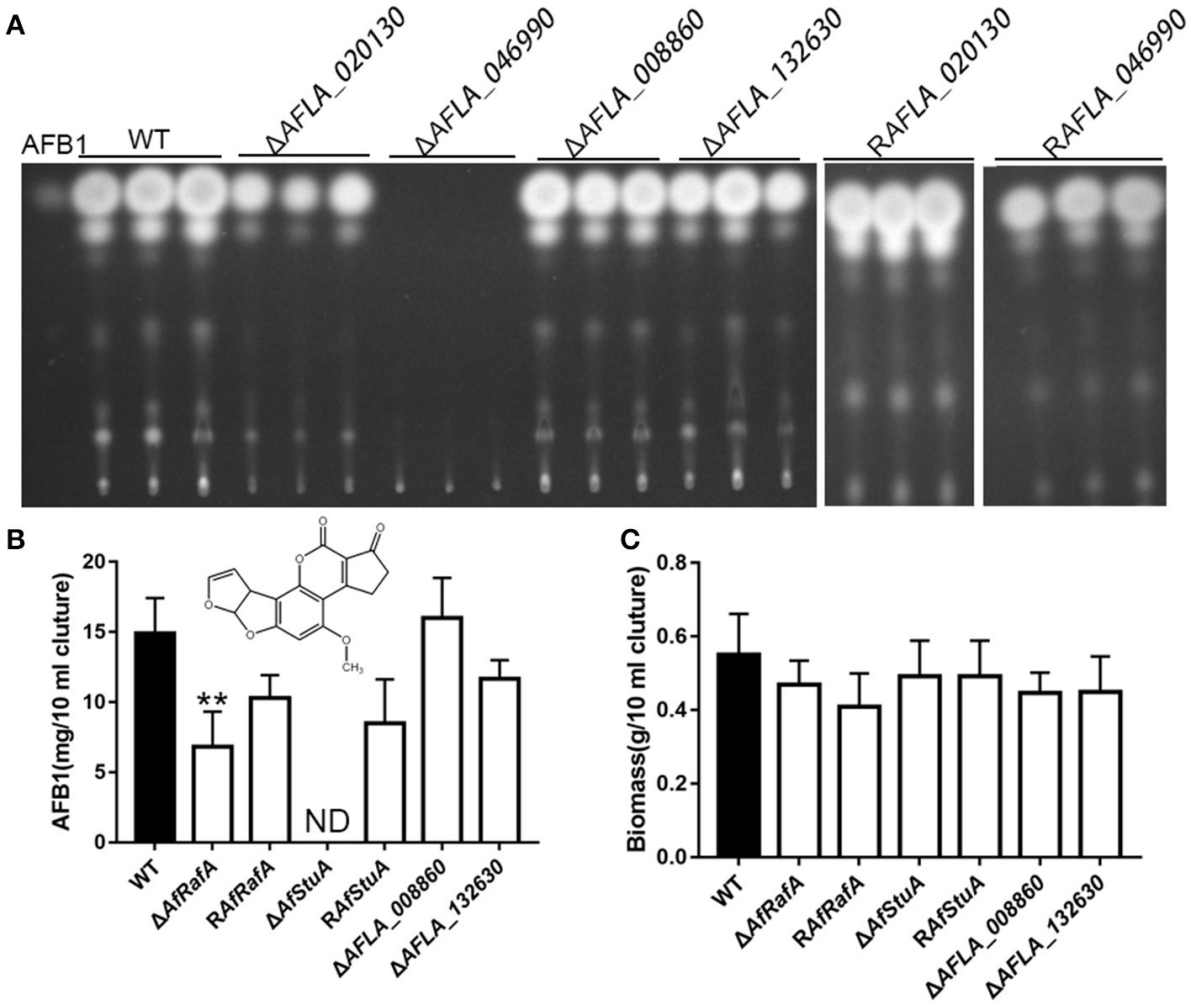
Aflatoxin production of APSES transcription factor deletion mutants. **(A)** Production of aflatoxin for each APSES TF disruptant, complementation, and WT strains in YES media were determined via TLC, and three biological repeats were performed in this experiment. ST represents 0.5 ug AFB1 standard. **(B)** HPLC quantification of AFB1 produced from all strains. **(C)** Fungal biomass of APSES TF disruptant, complementation, and WT strains. ND means could not be detectable. Three biological repeats were performed in these experiments. ^**^p ≤ 0.01.

To determine the particular roles of APSES TFs in aflatoxin synthesis, knock-out strains were generated based on homologous recombination. At least two deletion transformants for the genes *AFLA_020130, AFLA_046990, AFLA_008860*, and *AFLA_132630* were obtained, respectively. Unfortunately, we could not obtain the homozygous disruptant of gene *AFLA_076560* in the *A. flavus* PTSΔ*ku70*Δ*pryG* background even after two rounds of genetic transformation. Then, the aflatoxin production was determined for all deletants and the WT strain under YES media. As shown in Figure 2, loss of AFLA_020130 resulted in a dramatic reduction of AFB1 production compared to WT (Figure 2A), which was approximately half of that of WT (Figure 2B). We designated AFLA_020130 as AfRafA (an *A. flavus* regulator for aflatoxin synthesis) and AFLA_046990 as AfStuA (an *A. flavus* homolog of StuA). Most notably, Δ*AfStuA* produced no detectable AFB1 after 7 days while the concentration for the parental strain was ~15 mg per 10 ml of cultures (Figure 2B). Deletion of *AFLA_008860* or *AFLA_132630* appeared to have no effect on aflatoxin synthesis in *A. flavus*. Therefore, our subsequent work focused solely on the transcription factors AfRafA and AfStuA. Firstly, we verified the gene deletions of *AfRafA* and *AfStuA* in Δ*AfRafA* and Δ*AfStuA*, respectively, using a combination of PCR and Southern blot (Figures S1, S2). To clarify their function in *A. flavus*, gene complementation strains for Δ*AfRafA* and Δ*AfStuA* in the deletion strain background were generated. Furthermore, the RT-PCR verification for both deletion and complementation strains confirmed that the entire coding regions for both *AfRafA* and *AfStuA* had indeed been deleted in Δ*AfRafA* and Δ*AfStuA*, and were re-obtained in their complementation strains R*AfRafA* and R*AfStuA*, respectively (Figures S1, S2). Furthermore, both complementation strains restored the aflatoxin synthesis to the WT level (Figure 2). In addition, we also tested whether the observed reduced or complete lack of aflatoxin production in Δ*AfRafA* and Δ*AfStuA* were a result of their generally poor and slower growth. Clearly, as illustrated in Figure 2C, both Δ*AfRafA* and Δ*AfStuA* produced the same level of biomass as both WT or complementation strains. To further confirm that the defect in aflatoxin synthesis was caused via deletion of *AfRafA* and *AfStuA*, we also performed a toxin assay in the glutamine-containing medium, which was previously reported to induce aflatoxin production (Wang et al., 2017). Similar to YES, the results showed that loss of AfRafA reduced the toxin production, while deletion of AfStuA resulted in no detectable aflatoxin in glutamine media (Figure S3). In summary, our data corroborated that AfRafA was critically required, and further AfStuA was essential to activate aflatoxin biosynthesis.

### AfRafA and AfStuA are critically involved in fungal growth and conidial development

**Figure 3:**
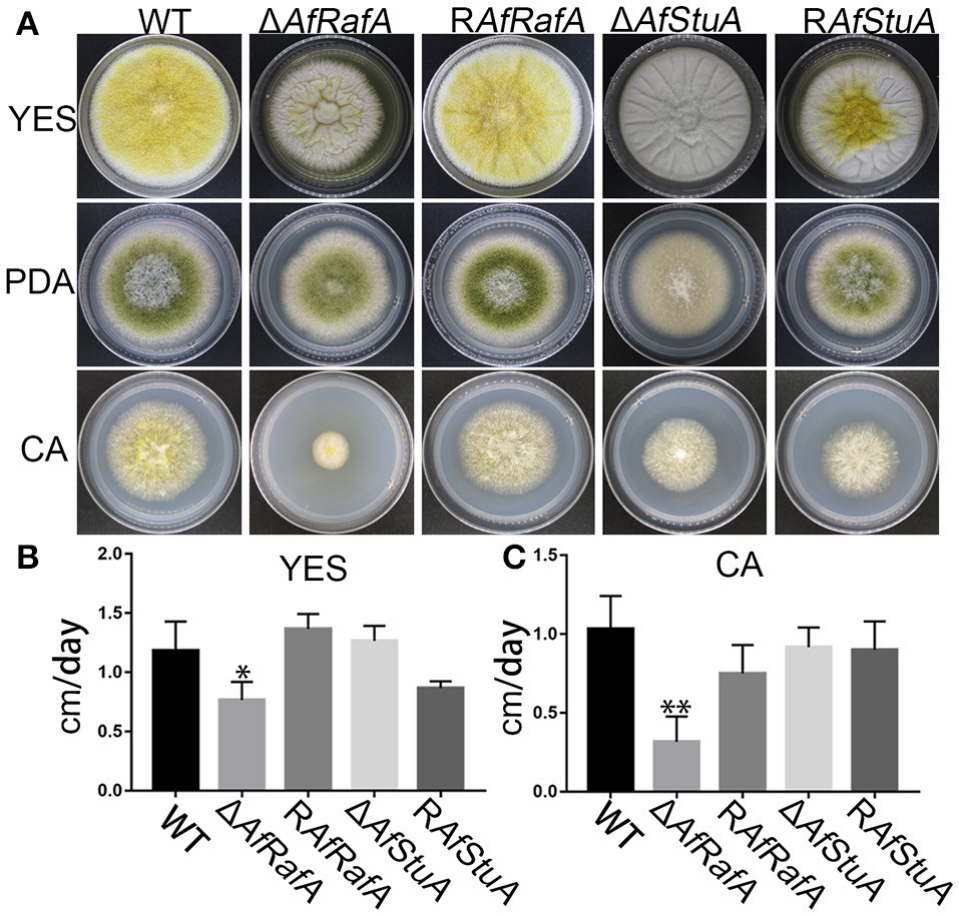
Defects of Δ*AfRafA* and Δ*AfStuA* mutants in fungal vegetative growth. **(A)** Colonies for gene deletion (Δ*AfRafA* and Δ*AfStuA*), complementation strains (R*AfRafA* and R*AfStuA*), and WT (CA14) cultured on YES, potato dextrose agar (PDA), and Czapek-Dox agar (CA) under 29°C for 6 days. **(B)** The growth rates of gene deletion and complementation strains on YES agar. **(C)** Growth rate of all strains on CA agar. We performed this experiment twice and three biological repeats were performed for each replicate. ^*^p ≤ 0.05, ^**^p ≤ 0.01.

**Figure 4:**
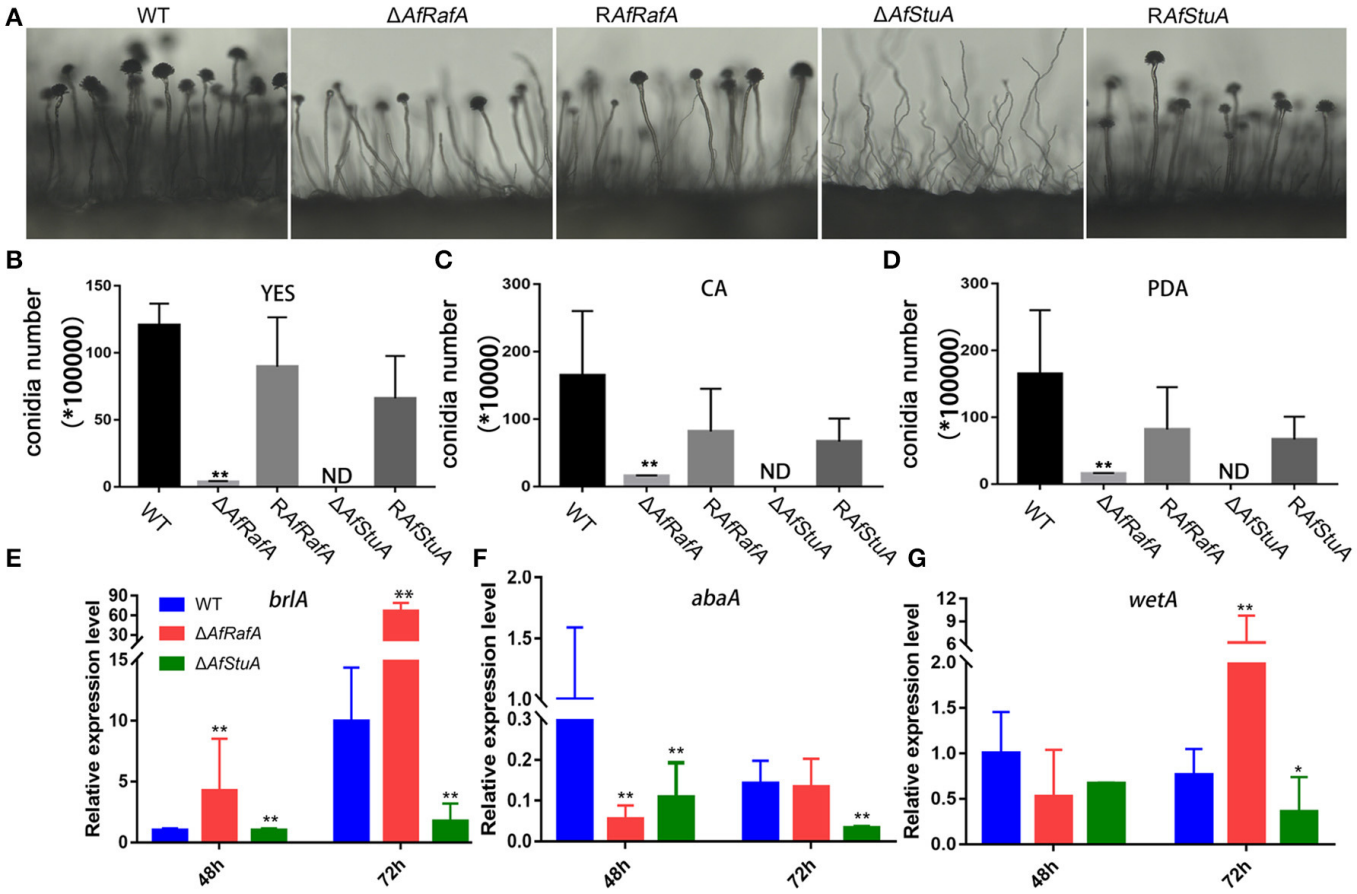
Defects of Δ*AfRafA* and Δ*AfStuA* mutants in fungal conidiation. **(A)** Colonies of Δ*AfRafA*, Δ*AfStuA*, R*AfRafA*, R*AfStuA*, and WT were pre-cultured on PDA and were transferred on sterile slides under 37°C overnight to induce conidiophores. Quantification of conidia produced by Δ*AfRafA*, Δ*AfStuA*, R*AfRafA*, R*AfStuA*, and WT on YES **(B)**, CA **(C)**, and PDA **(D)**. Expression of conidial marker genes *brlA* **(E)**, *AbaA* **(F)**, and *WetA* **(G)** in Δ*AfRafA*, Δ*AfStuA*, and WT on YES at 48 and 72 h. Error bars represent *SD*. Statistically significant difference, ^*^p ≤ 0.05, ^**^p ≤ 0.01. ND means could not be detected. We performed this experiment three times and three biological repeats were performed per experiment.

To explore the effect of APSES TFs on fungal morphogenesis and asexual development, the deletion, complementation, and WT strains were cultured on Czapek–Dox (CA), YES, and PDA media. Δ*AfRafA* and Δ*AfStuA* exhibited severe defects in fungal morphogenesis or conidiation (Figures 3, 4). Δ*AfRafA* formed fewer aerial hyphae on CA agar compared to WT (Figure 3A). The hyphal extension rate of Δ*AfRafA* was significantly slower than WT on YES (Figure 3B), and far more pronounced on CA (Figure 3C). As a result, after 6 days, the Δ*AfRafA* colony was smaller than its WT counterpart (Figure 3A). However, the germination rate of Δ*AfRafA* conidia was essentially identical to WT in YES or PDB (data not shown), suggesting that the slow growth was not a result of delayed germination. Microscopic analysis showed that under YES lipid medium, Δ*AfRafA* could form a conidiophore, but at a greatly reduced number (Figure 4A). As a consequence, Δ*AfRafA* was greatly reduced by 100 to 1,000-folds in conidia production when compared to WT (Figures 4B–D), and the conidial phenotype was most defective on YES (Figures 3A, 4B). The complementation strain R*AfRafA* fully restored the defective phenotypes that have been described above to WT level (Figures 3, 4). These results suggested that AfRafA played important roles in both vegetative growth and asexual development of *A. flavus*. Interestingly, although loss of AfStuA did not affect the colony growth of *A. flavus* (Figure 3), no conidia could be detected in Δ*AfStuA* under any of the three cultures assayed (Figures 3, 4). It is well-known that light triggers sporulation in most of the filamentous fungi. However, even under light, Δ*AfStuA* was still unable to sporulate (Figure S4A). In sharp contrast, the WT strain developed more conidia in light than in the dark. Furthermore, it was clear that Δ*AfStuA* lost the capacity to develop a normal conidiophore (Figure 4A). Previous reports found that adjoining a WT strain could rescue the non-sporulation phenotype in mutants such as FluG and VeA (Lee and Adams, 1994; Yager et al., 1998). However, this was not the case for AfStuA, as indicated by the undetectable conidiation when Δ*AfStuA* grew near the WT under either 29 or 37°C (Figure S4B). The *AfStuA* complementation strain re-obtained the ability to form conidiophores and produce WT-level conidia (Figures 3, 4). In summary, our observations indicate that AfRafA was required for both normal fungal vegetative growth and for asexual development, and that AfStuA was essential for inducing conidiophore formation and subsequent conidia development in *A. flavus*.

It has previously been reported that three transcription factors BrlA, AbaA, and WetA define a central regulatory pathway, modulating the sporulation process (Park and Yu, 2012), and thus work as a marker of conidiation-specific gene expression in the *Aspergillus* genus. To further explore the roles of AfRafA and AfStuA in the asexual development of *A. flavus*, the expression levels of these genes were examined in all strains. As shown in Figure 4E, expression of *brlA* and *wetA* was significantly downregulated (>2-fold at least at one timepoint) and *abaA* expression decreased by about 7- and 4-fold at 48 and 72 h in Δ*AfStuA* relative to WT, suggesting that lowering the expression level of three conidiation-essential genes was responsible for the development of the non-conidiation phenotype of Δ*AfStuA*. In contrast, expression of *brlA* increased by 4- and 7-fold in Δ*AfRafA* at 48 and 72 h, respectively, and expression of *wetA* had also greatly increased at 72 h (Figure 4G), albeit the downregulated expression of *abaA* occurred only at 48 h in Δ*AfRafA* relative to WT (Figure 4F). Therefore, we proposed that the conidial defects in Δ*AfRafA* were mainly caused by the abnormal expression of these three essential conidial genes. Unsurprisingly, expression of *brlA* via strong amylase gene promoter in *A. oryzae* has been reported to lead to an abnormal conidiation phenotype (Yamada et al., 1999). Upregulated expression of *brlA* and *abaA* in Δ*mpkB* also reduced the conidiation on MT media in *A. nidulans* (Kang et al., 2013). In summary, AfStuA and AfRafA likely control the conidial process by modulating downstream targets (BrlA-AbaA-WetA) and they do so either via down-regulation or misscheduled expression.

### AfStuA is absolutely required for sclerotia formation, and AfRafA plays a role in sclerotial size determination

**Figure 5:**
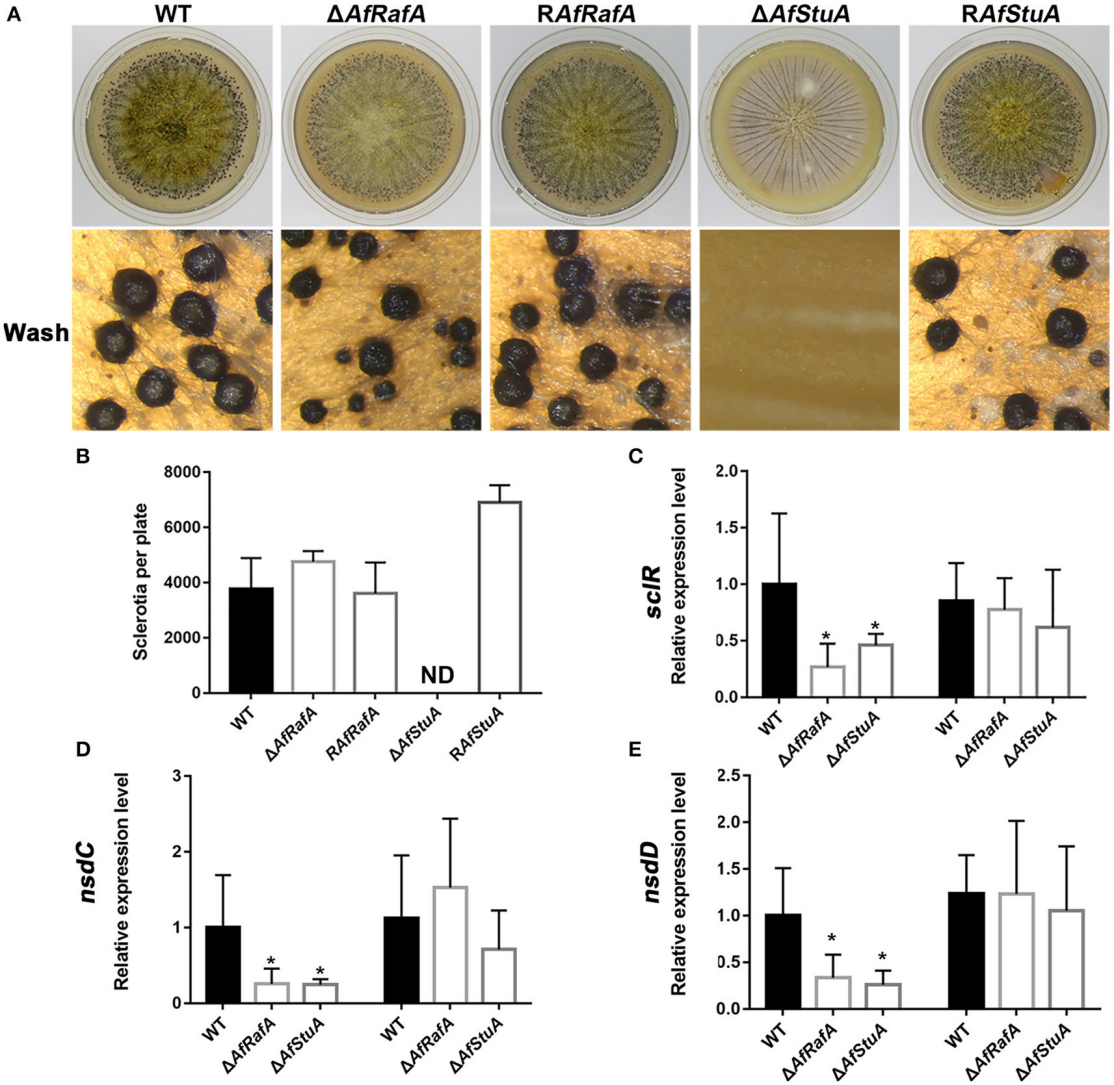
AfStuA was essential for sclerotial formation, and AfRaf was involved in sclerotial development. **(A)** Strains of Δ*AfRafA*, Δ*AfStuA*, R*AfRafA*, R*AfStuA*, and WT were cultured on WKM agar for 10 days to induce sclerotial formation. **(B)** Quantification of sclerotia in Δ*AfRafA*, Δ*AfStuA*, R*AfRafA*, R*AfStuA*, and WT cultured on WKM. Expression of sclerotial marker genes *sclR* **(C)**, *nsdC* **(D)**, and *nsdD* **(E)** in Δ*AfRafA*, Δ*AfStuA*, and WT on YES at 48 and 72 h. Error bars represent the *SD*. Statistically significant difference, ^*^p ≤ 0.05, ^**^p ≤ 0.01. ND means could not be detected. We performed this experiment three times and three biological repeats were performed per experiment.

The sclerotium functions as a resistant structure and protects fungi against various damages from environmental extremes and fungivores in their niches and thus, the sclerotium has an important role in pathogen propagation and proliferation. To evaluate the effects of AfRafA and AfStuA on sclerotium formation, Δ*AfRafA*, Δ*AfStuA*, complementation strain, and WT strain were inoculated on WKM medium to induce sclerotium development (Figure 5). Our observations showed that although Δ*AfRafA* produced the same number of sclerotia as both WT or the complementation strain, the size distribution of sclerotia distinctly differed from that of WT and the average size of Δ*AfRafA* (average size: 0.18 mm) was significantly smaller (*p* ≤ 0.05) than that of WT (average size: 0.286 mm) or the complementation strain (average size: 0.284 mm). However, the sclerotia of Δ*AfRafA* appeared to have the same sensitivity to heat or UV stress as WT (data not shown). In sharp contrast to the abundant sclerotia formed in WT, no sclerotia were observed in Δ*AfStuA*, and R*AfStuA* could rescue the defects, suggesting AfStuA to be fatal for sclerotial development. To further clarify the effects of AfRafA and AfStuA on sclerotial development, expression of three regulators SclR, NsdC, and NsdD encoding genes, which control the sclerotial development process (Jin et al., 2011; Cary et al., 2012), were therefore tested and are displayed in Figure 5. Expression of all of three important activator genes *NsdC, NsdD*, and *SclR* were downregulated by >2-fold in Δ*AfRafA* and were even further downregulated in Δ*AfStuA* at 48 h, which was consistent with the reduction and missing of sclerotia formation in the respective mutants. In summary, these results suggested AfStuA as an essential regulator for sclerotia formation, and AfRafA to play a significant role for the size determination during sclerotial development.

### AfRafA and AfStuA are central regulators for activating the aflatoxin gene cluster expression, and AfStuA functions upstream of the pathway-specific transcription factor AflR

**Figure 6:**
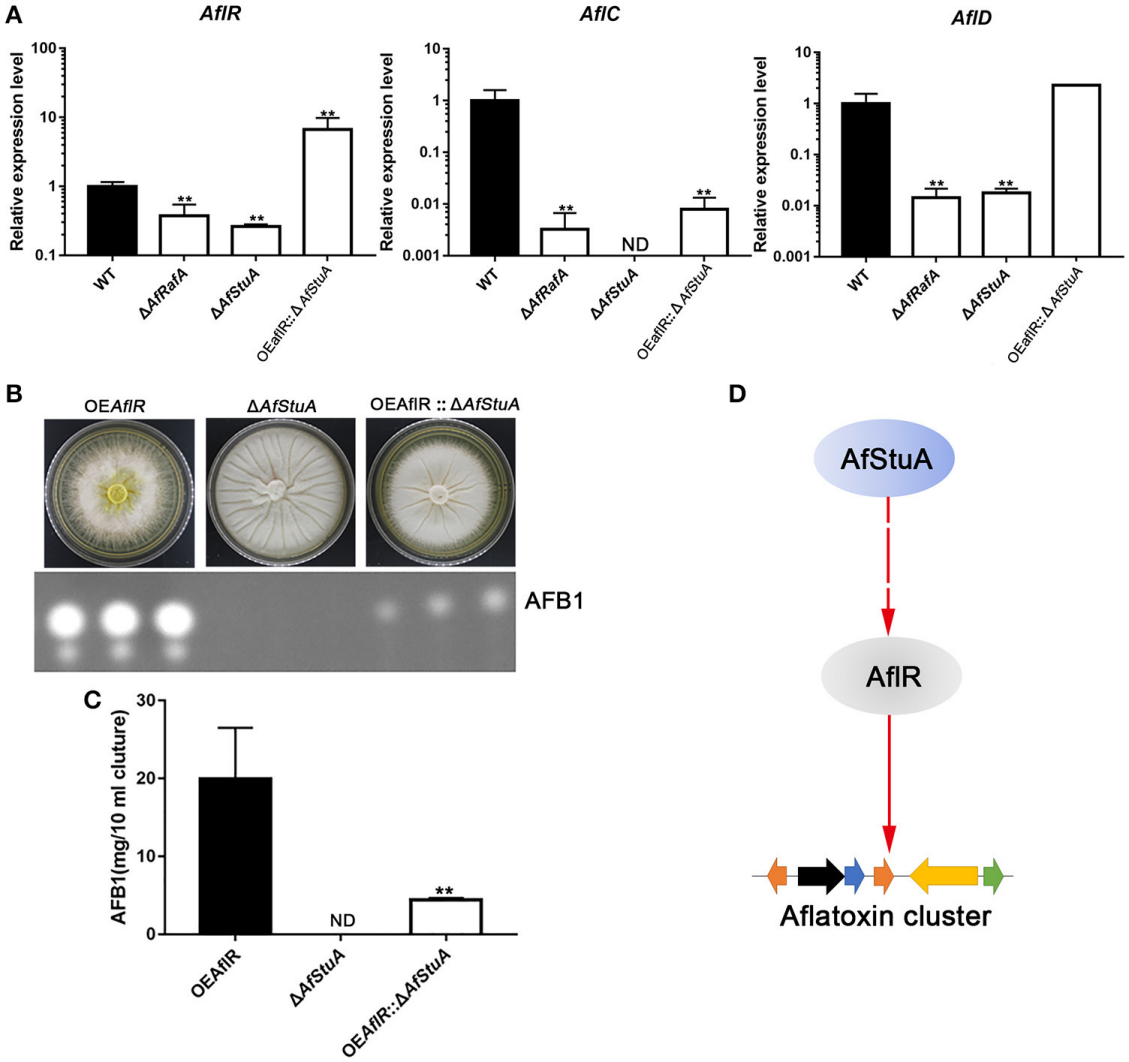
AfRafA positively regulated aflatoxin cluster expression and furthermore, AfStuA played an essential role in partially activating the aflatoxin expression via AflR. **(A)** The transcript abundance of aflatoxin cluster genes *AflR, AflC*, and *AflD* in Δ*AfRafA*, Δ*AfStuA*, OEAflR:: Δ*AfStuA*, and WT under YES media for 48 and 72 h, determined via qPCR. **(B)** Determination of AFB1 in OEAflR, Δ*AfStuA*, and OEAflR:: Δ*AfStuA*. **(C)** Quantification of AFB1in OEAflR, Δ*AfStuA*, and OEAflR:: Δ*AfStuA*. **(D)** Working model for the regulatory pathway of AfStuA in the aflatoxin cluster expression. Error bars represent *SD*. Statistically significant difference, ^*^p ≤ 0.05, ^**^p ≤ 0.01. ND means could not be detected.

To determine whether AfStuA and AfRafA regulate aflatoxin production at a transcription level, expression of the aflatoxin gene cluster was evaluated via qPCR in their deletants and WT. As expected, expressions of aflatoxin cluster genes (*aflR, aflC*, and *aflD*) were significantly downregulated in absence of *AfRafA*, and even lower or completely missing in Δ*AfStuA* as compared to WT (Figure 6A). Expression of the aflatoxin transcription activator encoding gene *aflR* decreased by 2.6-fold in Δ*AfRafA* and by 3.8-fold in Δ*AfStuA. aflC*, which encodes the polyketide synthase that is responsible for aflatoxin skeleton synthesis, was more than 100-fold reduced in Δ*AfRafA*. In particular, its expression was completely blocked in the absence of AfStuA (Figure 6A), which could explain the complete loss of aflatoxin in Δ*AfStuA* (Figure 2). Also, the expression level of *aflD*, whose protein product was responsible for converting norsolorinic acid to versicolorin (Cary et al., 1996), was downregulated more than 50-fold in Δ*AfRafA* and Δ*AfStuA* compared to that of WT (Figure 6A). AflR is pathway-specific transcription factor, which are responsible for inducing AFB gene cluster expression; consequently, loss of AlfR resulted in silencing of most genes and a complete stop of toxin production (Yu et al., 1996). Considering that only Δ*AfStuA* (but not Δ*AfRafA*) resembled the Δ*aflR* mutant with respect to the phenotype of aflatoxin biosynthesis (lack of expression for the aflatoxin cluster gene and lack of toxin production), it is therefore reasonable to suspect that AfStuA might control the aflatoxin expression via an AflR-dependent pathway. To verify this hypothesis, a OE*AflR:*: Δ*AfStuA* was engineered where *aflR* was over-expressed in combination with *AfStuA* deletion. Interestingly, as the aflatoxin synthesis was restored in OE*AflR:*: Δ*AfStuA* (Figure 6B), the AFB1 level (4.4 ± 0.2 mg/10 ml cultures) still remained significantly below that of WT (14.9 ± 2.5 mg/10 ml cultures mg), or OE*AflR* (19.9 ± 6.6 mg/10 ml cultures) (Figures 2B, 6C). As expected, OE*AflR:*: Δ*AfStuA* could not develop conidia with a phenotype similar to the Δ*AfStuA* mutant (Figure 6B). In fact, expression of *aflR* in OE*AflR:*: Δ*AfStuA* was >7-fold upregulated relative to WT (Figure 6A). As a consequence, the mRNA level of *aflD* was restored to WT-level. However, expression of *aflC* was still >80-fold lower in OE*AflR:*: Δ*AfStuA* than in WT (Figure 6A), which could explain the unrecovered aflatoxin production (Figure 6B). In summary, these results suggest that AfStuA controls the aflatoxin synthesis via at least partial modulation of the AflR expression (Figure 6D), while further unidentified proteins, functioning downstream of AfStuA, might be required to fully activate aflatoxin synthesis. It is well-known that LaeA is a global regulator for many SM, which are essentially required for aflatoxin cluster gene expression together with the VeA (Kale et al., 2008; Cary et al., 2015). To further study the regulatory mechanisms underlying AfStuA and AfRafA, the expressions for *laeA* and *veA* were examined (Figure S5). Our results show that no significant difference exists between Δ*AfRafA* or Δ*AfStuA* vs WT, indicating that AfRafA and AfStuA modulated aflatoxin synthesis independent of the LaeA/VeA complex. In addition, we also evaluated the expression of the *AfmtfA* gene between mutants and WT, which is a novel transcription factor that was recently found to be critical for aflatoxin biosynthesis (Zhuang et al., 2016). Down-regulation of *AfmtfA* by approximately half was observed in Δ*AfRafA* and Δ*AfStuA*, implying that it might serve as a potential downstream target of AfRafA and AfStuA, mediating the regulatory pathway in aflatoxin synthesis. In summary, AfStuA regulates aflatoxin in an AflR-dependent manner, irrespective of the LaeA/VeA complex.

### AfStuA and AfRafA play critical roles in the fungal colonization of plant seeds

**Figure 7:**
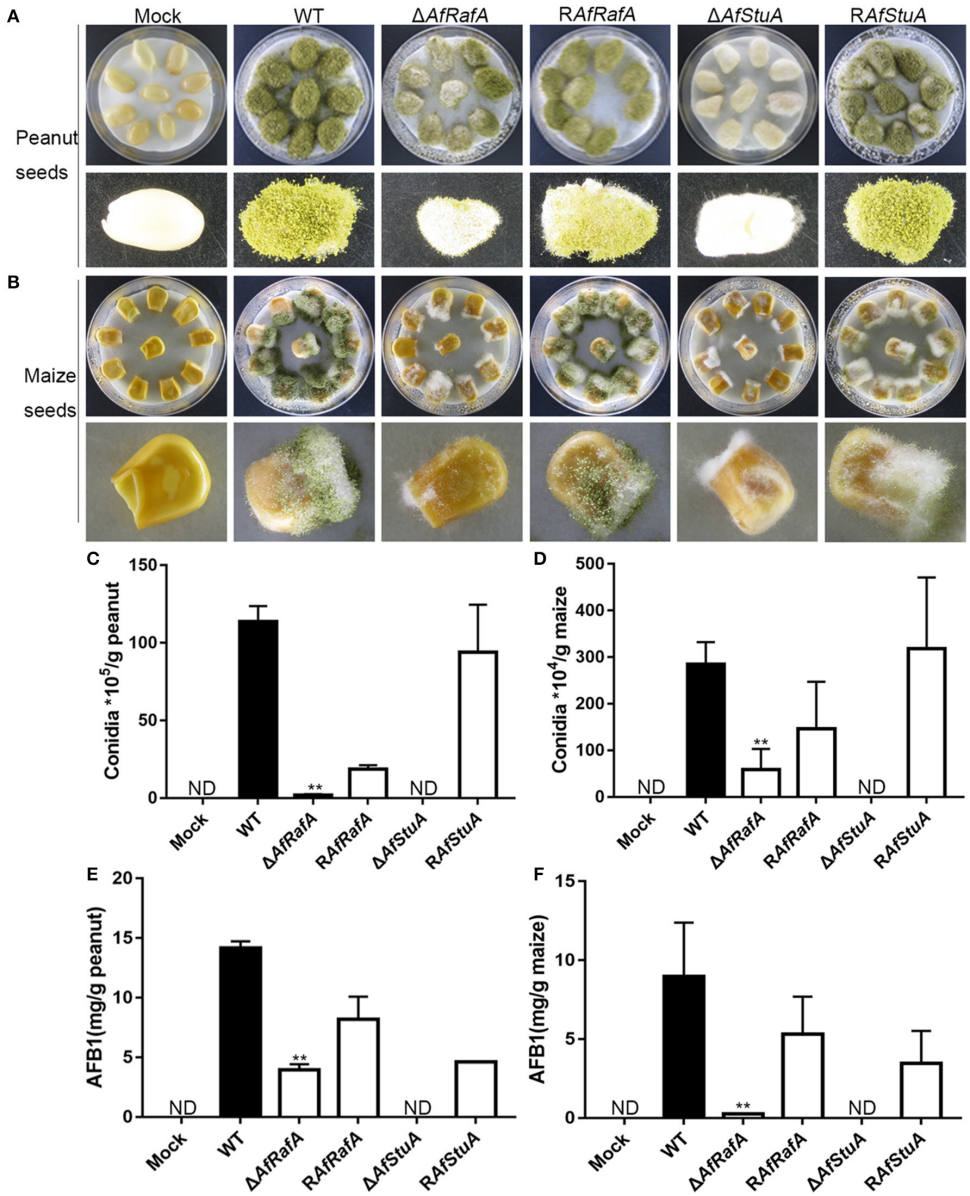
Δ*AfRafA* and Δ*AfStuA* were impaired in the colonization of plant seeds. Infection of peanut cotyledons **(A)** and maize seeds **(B)** after culture under 29°C for 6 days with a sterile and wet filter paper to retain high humidity. **(C)** Quantification of conidial production in infected peanut seeds. **(D)**. Quantification of the conidial production in infected maize seeds. **(E)** Quantification of AFB1 in infected peanut seeds via HPLC. **(F)** Quantification of AFB1 in infected maize seeds. Error bars represent *SD*. Statistically significant difference, ^**^p ≤ 0.01. ND could not be detected.

Based on these results of Δ*AfRafA* and Δ*AfStuA* exhibiting a variety of defects in vegetative growth, conidial and sclerotial development, and biosynthesis of the secondary metabolite aflatoxin, we propose that both proteins might play roles in the colonization of plant seeds by *A. flavus*. To determine the functions of AfStuA and AfRafA for fungal pathogenesis, we used peanut and maize seeds, which represented the main staples to frequently carry AFB1, to perform the infection assays. The surfaces of both peanut and maize seeds were sterilized and then inoculated with *A. flavus* strains, including Δ*AfRafA*, Δ*AfStuA*, R*AfRafA*, R*AfStuA*, and WT. Not inoculated plant seeds were used as control (Mock). It was apparent that both Δ*AfRafA* and Δ*AfStuA* displayed severe defects for the colonization of plant seeds (Figure 7). More specifically, the colonization of plant seeds by Δ*AfRafA* or Δ*AfStuA* was delayed, and only little or sparse mycelia could be observed on the surface of peanut and maize seeds for each gene deletion mutant compared to both WT or complementation strains (Figures 7A,B). As a result, significantly reduced fungal biomass could be observed in Δ*AfRafA* and Δ*AfStuA* infected plant seeds relative to WT. In contrast to WT or complemented strains, both conidia and aflatoxin of Δ*AfRafA* were markedly reduced in response to growth on peanut and maize seeds, respectively (Figures 7C–F). Furthermore, no conidiation was observed for Δ*AfStuA* in response to growth on plant seeds (Figures 7C,D), which coincided with the results observed on various artificial media *in vitro* (Figures 3, 4 and Figure S4). The AFB1 levels on Δ*AfStuA* infected peanut and maize seeds were undetectable, which was in good agreement with the non-aflatoxigenic phenotype *in vitro* (Figure 2 and Figure S3), strongly demonstrating AfStuA function as a key regulating mechanism in aflatoxin synthesis both in artificially inducing media and *in planta*. The complementation strains R*AfRafA* and R*AfStuA* basically rescued all these defects in their respective deletion mutants (Figure 7). As expected, neither *A. flavus* conidia nor aflatoxin could be detected in the mock without infection with *A. flavus* (Figure 7). In summary, our data fully demonstrated that the two APSES transcription factors AfRafA and AfStuA play critical roles in the infection and colonization of plant seeds by *A. flavus*.

### AfRafA and AfStuA play antagonistic roles in cyclopiazonic acid production

**Figure 8:**
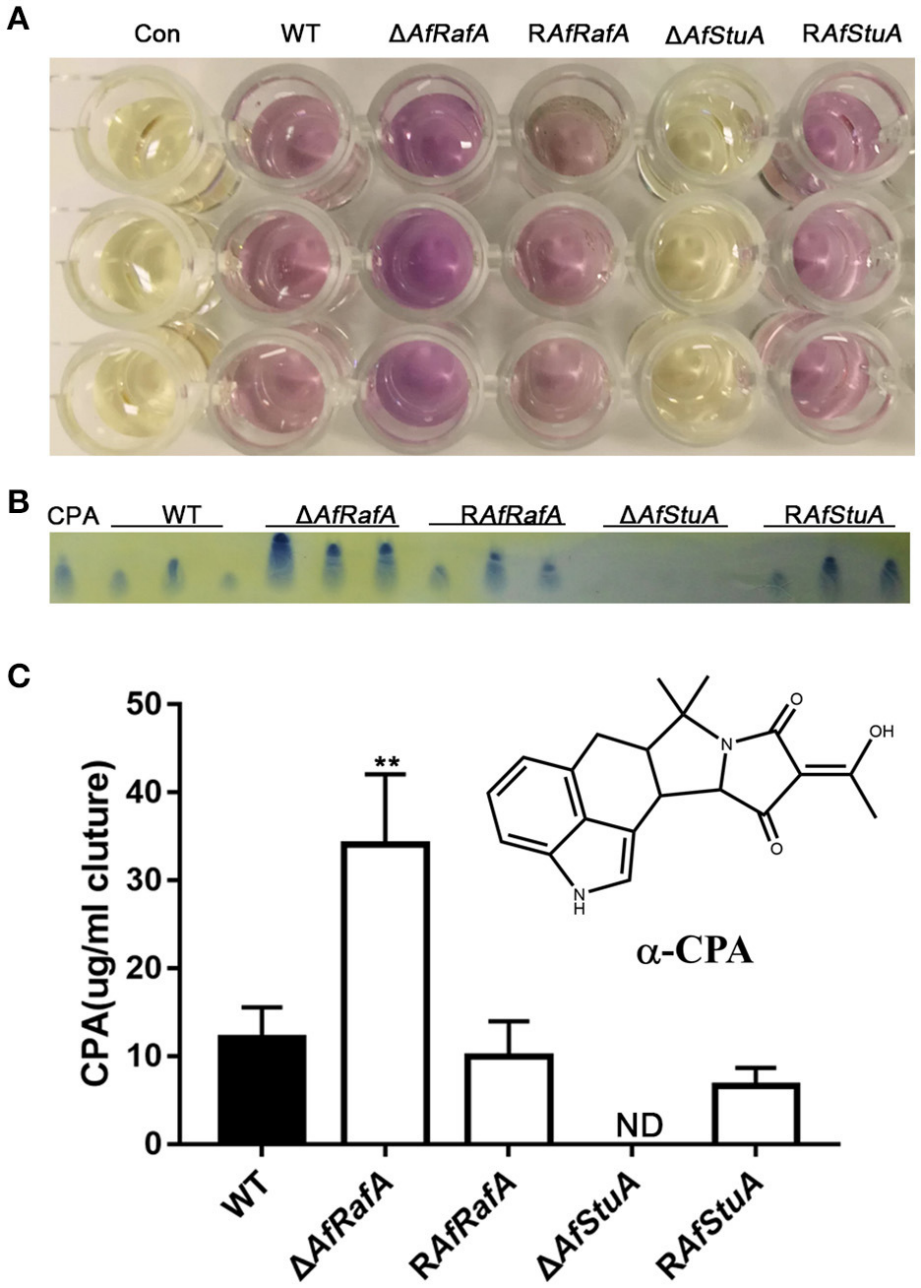
AfStuA is essential for inducing CPA synthesis, while AfRafA negatively regulated CPA biosynthesis. **(A)** Direct examination of the CPA production in cultures of Δ*AfRafA*, Δ*AfStuA* R*AfRafA*, R*AfStuA*, and WT growth in WKM. **(B)** TLC analysis of CPA concentration, where ST represents the CPA standard. **(C)** Quantification of CPA concentration. Error bars represent *SD*. Statistically significant difference, ^**^p ≤ 0.01. ND could not be detected. We performed this experiment three times and three biological repeats were performed per experiment.

CPA is another mycotoxin produced by *A. flavus*, which exerts its toxic effect via inhibition of the Ca^2+^-ATPase in the sarcoplasmic reticulum, ultimately enhancing muscular contractions (Goeger and Riley, 1989). Previous studies reported that five genes [*mfs1* (*AFLA_139470), maoA* (*AFLA_139470*), *dmaT* (*AFLA_139480*), *pks-NRPS* (*AFLA_139490*), and *ctfR1* (*AFLA_139500*)] were assembled into the CPA cluster (Chang et al., 2009). To explore whether CPA synthesis were affected by AfRafA and AfStuA, we quantified the CPA production in Δ*AfRafA*, Δ*AfStuA*, the complementation of R*AfRafA* and R*AfStuA*, and WT. All strains above were cultured in WKM media at 29°C for 7 days. The CPA was directly and visually analyzed in cultures by adding the Ehrlich's reagent. Clearly, the color change of Δ*AfRafA* was more pronounced than that of WT (Figure 8A). However, no change was observed in Δ*AfStuA* cultures after reagent addition, as well as in the negative control without inoculation of *A. flavus* (Figure 8A). To quantitively analyze the CPA concentration, chemicals were extracted and analyzed via thin layer chromatography (TLC). As expected, the results showed that Δ*AfRafA* produced four-fold more CPA than WT, whereas Δ*AfStuA* produced no CPA (Figures 8B,C). Both complementation strains restored CPA synthesis to the WT level. In summary, these data confirmed that AfRafA negatively controlled, while AfStuA positively activated CPA biosynthesis.

## Discussion

### AfRafA and AfStuA play essential roles in aflatoxin synthesis

The APSES transcription factor family are unique to fungi and function as key regulators of cellular differentiation, vegetative growth, and asexual and sexual development from *S. cerevisiae* to plant or human pathogens as reviewed previously (Zhao et al., 2015). However, a systematic investigation of their roles in the regulation of the metabolism and the synthesis of mycotoxin was conducted for the first time in this study. Unfortunately, we could not obtain the genetic knockout mutant for *AFLA_076560*, implying that this gene is vital for *A. flavus*. An important finding was that two of the five *A. flavus* APSES proteins (AfRafA and AfStuA in particular), have been demonstrated to be essential for both development and biosynthesis of SMs of *A. flavus*. Most strikingly, disruption of AfStuA resulted in silencing of aflatoxin cluster genes. Consequently, no aflatoxin could be detected in Δ*AfStuA in vitro*, or in its colonization of plant seeds. Furthermore, our results demonstrated that AfStuA modulated aflatoxin gene cluster activation in a pathway-specific transcription factor AflR dependent way, which uncovered a novel hierarchy in the complex regulatory network. However, overexpression of AflR only partly restored the aflatoxin synthesis in Δ*AfStuA*. Using glutamine media, overexpression of *aflR* could not rescue the aflatoxin production in Δ*AfStuA*, suggesting that AfStuA may control aflatoxin biosynthesis mediated by nitrogen metabolism. Therefore, it is interesting to both explore and define the roles of other unknown targets (downstream of AfStuA) in the regulation of this secondary metabolite. The LaeA-VeA-VelB complex plays a central role in activating the expression of the secondary metabolism in filamentous fungi (Bayram et al., 2008). However, the expressions of *LaeA* and *VeA*, which have previously been reported to regulate aflatoxin also by activating AflR, remained unaffected in Δ*AfRafA* or Δ*AfStuA*. Here, two possible cases are conceivable: (1) LaeA and VeA function upstream of AfStuA and AfRafA, which is supported since the expression level of AfStuA was significantly affected in Δ*AfVeA* (Cary et al., 2015). (2) AfStuA and AfRafA mediated a novel regulatory cascade, which is independent of LaeA/VeA, when considering that Δ*AfStuA* was more defective than Δ*AfLaeA* or Δ*AfVeA* in conidia, sclerotia, and aflatoxin gene expression (Kale et al., 2008). Expression of AfRafA was unaffected by the absence of AfStuA, and vice versa (data not shown), indicating that AfRafA and AfStuA mediate two independent regulatory pathways that induce aflatoxin expression.

### Both AfRafA and AfStuA are critically involved in fungal development

Our results demonstrated that Δ*AfStuA* could not develop conidiophores, and subsequently conidia under various assayed inducing conditions, thus confirming its vital role in the fungal asexual development, which was consistent with previous reports for many filamentous fungi (Zhao et al., 2015). The transcription factors BrlA, AbaA, and WetA constituted the central regulatory pathway, which is essential for the asexual development of fungi of the *Aspergillus* and *Penicillium* genera. It has been reported that the expression of all three transcription factor genes dramatically decreased in Δ*AfStuA* in both qPCR analysis. Furthermore, coupled with the DNA binding sites of StuA, which were identified in their promoters, a model was supported that was previously proposed for *A. nidulans* (Wu and Miller, 1997). However, expression of FluG encoding genes, which are responsible for secreting the conidiation-inducing factor and five development activators (FlbA, FlbB, FlbC, FlbD, and FlbE), functioning upstream of BrlA, remained almost unaffected in Δ*AfStuA* (data not shown). In summary, we propose that StuA may function as a novel central node, separate from the canonical model (Park and Yu, 2012), which also targeted the BrlA-mediated central regulatory pathway in fungal asexual development. Roles of AfRafA in conidial and sclerotial development were identified in *A. flavus* for the first time. Interestingly, *brlA* was up-regulated in Δ*AfRafA* and consequently, *wetA* was also increased at the later stage. These alterations led to delay and reduction in conidiation, implying that proper expression of central regulators (BrlA, AbaA, and WetA) was required for the normal fungal asexual development of *A. flavus*. This was also supported by a previous study (Yamada et al., 1999).

### AfRafA and AfStuA play opposite roles in the regulation of biosynthesis of CPA

The further interesting finding was that CPA was significantly upregulated in Δ*AfRafA*, whereas it was completely blocked in Δ*AfStuA*. Although, aflatoxin and CPA were frequently co-occurring in crops and bioproducts and CPA gene cluster are next to the aflatoxin cluster, *A. flavus* adopted different molecular mechanisms to control the activation of both mycotoxins. More specifically, AfStuA stimulates the biosynthesis of both CPA and aflatoxin, while AfRafA activates CPA, but inhibits the aflatoxin cluster. Our data would provide valuable information to enable a full understanding of the complex regulatory mechanism for mycotoxin biosynthesis in *A. flavus*.

In conclusion, this study performed a systemic screen for APSES TFs in the plant pathogenic fungus *A. flavus*. In a first, two APSES TFs were identified with overlapping and divergent functions; these are essential regulators for mycotoxin biosynthesis, including aflatoxin and CPA, conidiation, and pathogenesis. Our results provide further evidence for the association between fungal development and SM biosynthesis at the regulatory level. In addition, AfRafA, and especially AfStuA are potential novel targets for plant breeding or development of fungicides, considering their important roles in fungal development, mycotoxin synthesis, and pathogenicity, as well as their high level of conservation and confinement to fungi. Interestingly, it has recently been reported that gene silencing of *aflC* could effectively reduce aflatoxin in transgenic maize (Thakare et al., 2017), which provides a promising avenue for the removal of mycotoxin. Transcription factors generally govern a multitude of cellular processes, and therefore disruption of this regulator is often more effective to kill the pathogen than putative virulence targets. Regarding the AfStuA in *A. flavus* identified here, fungicide targeting could resolve two problems with the same intervention, (1) elimination of aflatoxin and other mycotoxin production, (2) blocking the development of conidia and sclerotia, thus avoiding secondary infection.

## Author contributions

GY, FZ, XN, JY, ZZ, XW, and SW conceived and designed the work. GY performed the experiments, analyzed the data, and wrote the manuscript. SW revised the manuscript.

### Conflict of interest statement

The authors declare that the research was conducted in the absence of any commercial or financial relationships that could be construed as a potential conflict of interest.

## Supplementary material

Figure S1 Verification of *AfRafA* deletion and complementation strain. The deletion and complementation of gene *AfRafA* were verified via PCR **(Up)**, Southern blot **(Middle)**, and RT-PCR **(Below)**.

Figure S2 Verification of *AfStuA* deletion and complementation strain. The deletion and complementation of gene *AfStuA* were verified via PCR **(Up)**, Southern blot **(Middle)**, and RT-PCR **(Below)**.

Figure S3 Determination of aflatoxin synthesis under GMM+glutamine media. All strains were cultured in GMM under addition of 5 mM glutamine media, and the produced aflatoxin was analyzed via TLC. The experiment was performed three times and three biological repeats were performed per experiment.

Figure S4 Light and near the WT could not induce Δ*AfStuA* conidiation. **(A)** Strains of WT, Δ*AfStuA*, and R*AfStuA* were cultured under light to induce conidia. **(B)** Δ*AfStuA* was cultured near the WT strain.

Figure S5 Expression of *laeA, veA*, and *mtfA* in Δ*AfRafA*, Δ*AfStuA*, and WT.

Table S1 Primers used in this study.
