# Supplementary figures and images for: Essential APSES Transcription Factors for Mycotoxin Synthesis, Fungal Development, and Pathogenicity in Aspergillus flavus
Source: Front Microbiol. 2017 Nov 20;8:2277. doi: 10.3389/fmicb.2017.02277 (PMC5702001; doi:10.3389/fmicb.2017.02277)

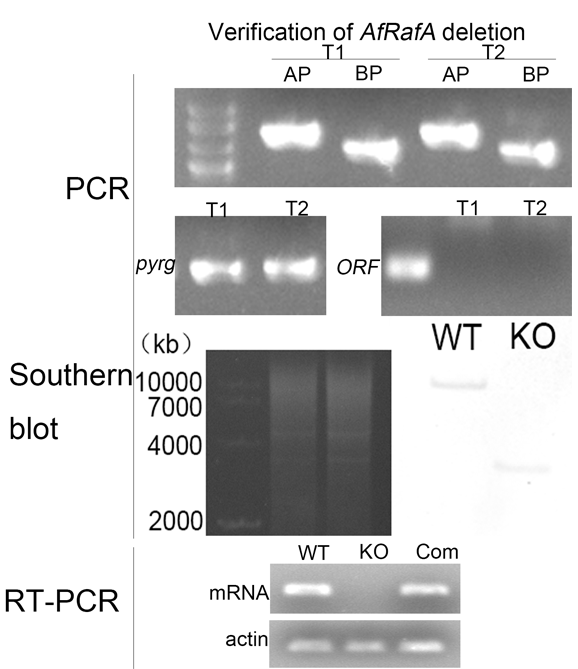

Supplement: Figure S1 — Verification of AfRafA deletion and complementation strain. The deletion and complementation of gene AfRafA were verified via PCR (Up), Southern blot (Middle), and RT-PCR (Below). [file Image1.TIF]

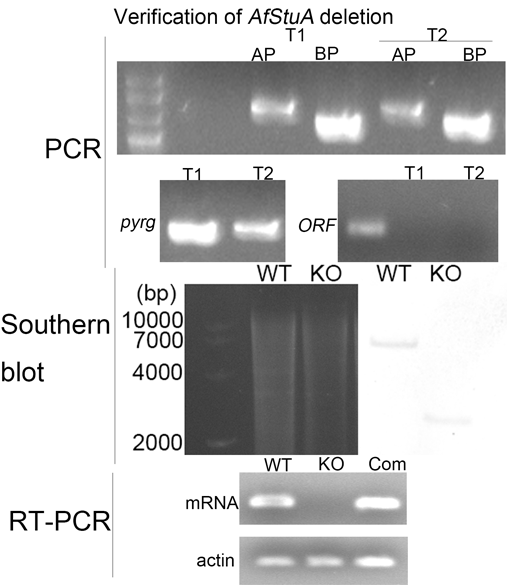

Supplement: Figure S2 — Verification of AfStuA deletion and complementation strain. The deletion and complementation of gene AfStuA were verified via PCR (Up), Southern blot (Middle), and RT-PCR (Below). [file Image2.TIF]

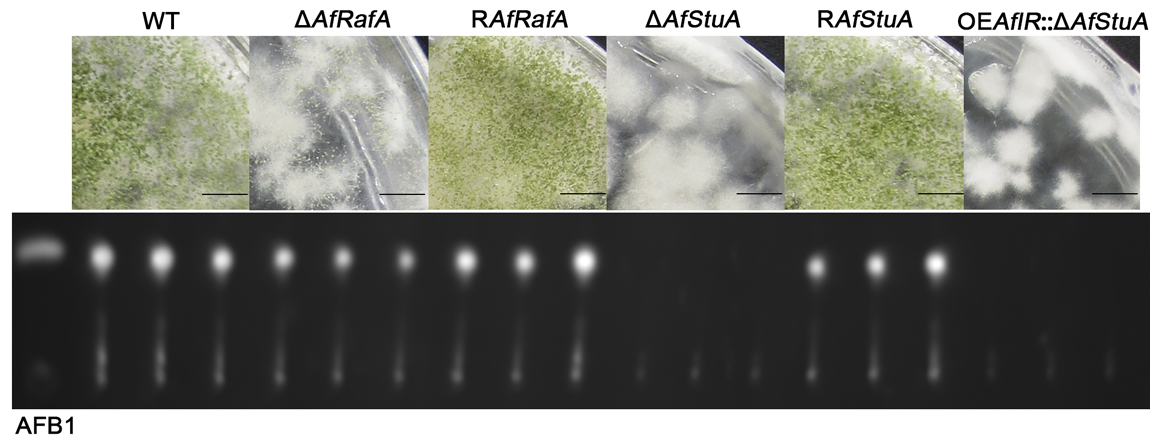

Supplement: Figure S3 — Determination of aflatoxin synthesis under GMM+glutamine media. All strains were cultured in GMM under addition of 5 mM glutamine media, and the produced aflatoxin was analyzed via TLC. The experiment was performed three times and three biological repeats were performed per experiment. [file Image3.TIF]

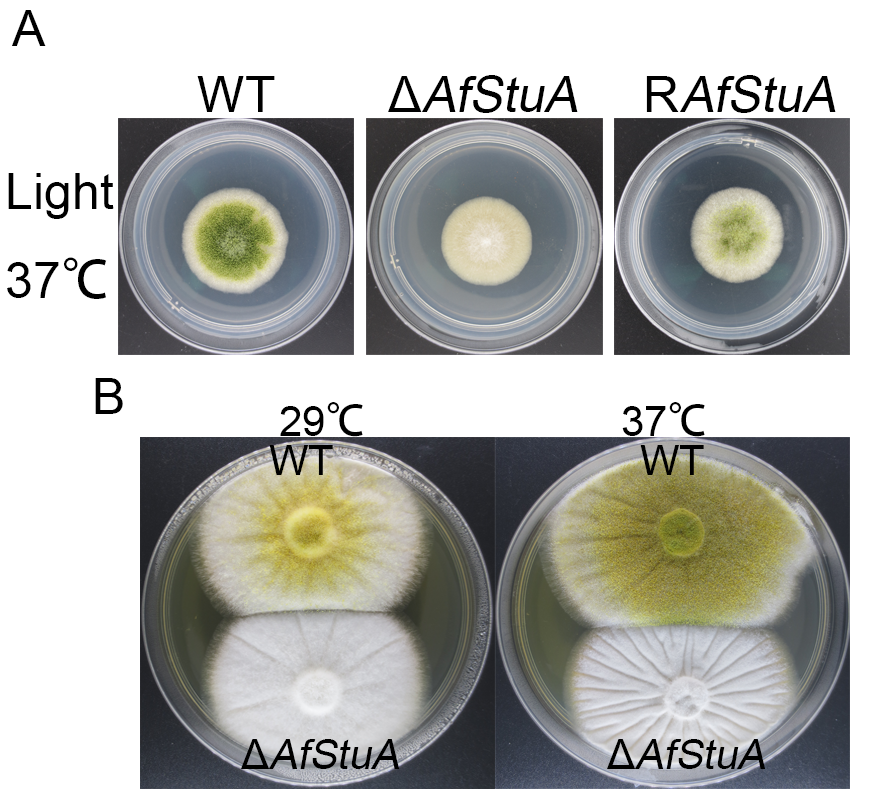

Supplement: Figure S4 — Light and near the WT could not induce ΔAfStuA conidiation. (A) Strains of WT, ΔAfStuA, and RAfStuA were cultured under light to induce conidia. (B) ΔAfStuA was cultured near the WT strain. [file Image4.TIF]

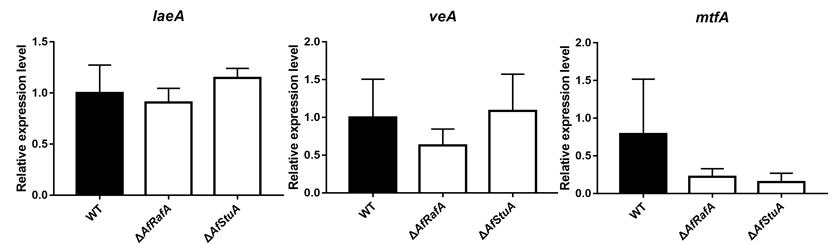

Supplement: Figure S5 — Expression of laeA, veA, and mtfA in ΔAfRafA, ΔAfStuA, and WT. [file Image5.TIF]
